# Evaluating the antifungal efficacy and bioactive metabolites of a traditional polyherbal aqueous formulation BioCC+ against Fusarium graminearum and Fusarium verticillioides

**DOI:** 10.1099/acmi.0.001173.v3

**Published:** 2026-08-11

**Authors:** Elisee Kouassi Kporou, Zilfa Irakoze, Nataliia Voloshchuk, Joshua D. Lambert, Joshua J. Kellogg, Josephine Wee

**Affiliations:** 1 Department of Food Science, The Pennsylvania State University, University Park, PA, USA; 2 Department of Biochemistry and Microbiology, Jean Lorougnon Guede University, Daloa, Côte d’Ivoire (Ivory Coast); 3 Department of Veterinary & Biomedical Sciences, The Pennsylvania State University, University Park, PA, USA; 4 One Health Microbiome Center, HUCK Institute for the Life Sciences, The Pennsylvania State University, University Park, PA, USA

**Keywords:** biochemometrics, biofungicide, fungal inhibition, *Fusarium *spp., metabolomics, mycotoxins

## Abstract

Fungal contamination of cereal crops threatens global food security by compromising grain quality and introducing harmful mycotoxins that endanger human and animal health. In West Africa, the long-standing ancestral use of native plant extracts to manage fungal contamination provides sustainable and locally adaptable solutions. Building on this knowledge, BioCC+, a polyherbal aqueous formulation containing *Azadirachta indica*, *Cymbopogon citratus*, *Allium sativum* and *Capsicum frutescens*, was developed to control maize contamination during storage in Côte d’Ivoire. This study evaluated the antifungal activity of BioCC+ against *Fusarium graminearum* and *Fusarium verticillioides* followed by bioactivity-guided untargeted metabolomics to explain observed effects. Crude BioCC+ extracts inhibited *F. graminearum* at 12.5 mg ml^−1^ and *F. verticillioides* at 25.0 mg ml^−1^, with the dichloromethane extract (DCMe) showing greater antifungal activity than methanol or water extracts. Based on the observed growth inhibition, DCMe was selected for fractionation using silica gel column chromatography with the following mixtures of hexane:ethyl acetate as the mobile phase: 100 : 0 for F1, 75 : 25 for F2, 50 : 50 for F3, 25 : 75 for F4 and 0 : 100 for F5, as well as 100% methanol for F6. Fractionation of DCMe resulted in three active fractions (F3, F4 and F6), with F4 showing the strongest inhibition (MIC=0.4 mg ml^−1^) against *F. graminearum*. Integration of antifungal activity assays with chemometric data through multivariate, univariate and correlation-based analysis identified six putative antifungal candidates, two known and four uncharacterized metabolites. These results provide a first step for integrating bioactivity assays with metabolomics to validate ancestral knowledge and develop community-driven practices for mitigating fungal contamination in staple crops.

## Data Summary

All data generated or analysed in this study are either presented in the manuscript or publicly accessible in the ScholarSphere data repository [1]. Raw spectral data for untargeted metabolomics are accessible on MassIVE.

**Impact Statement**

This study integrates antifungal bioassays with untargeted metabolomics to identify six putative metabolites that were prioritized as high-ranking candidates driving antifungal activity of a prototype biofungicide derived from ancestral plant mixtures from Côte d’Ivoire. By validating traditional practices with culture-based microbiology and modern biochemometrics, our findings support the development of sustainable, locally adapted antifungal solutions for reducing mycotoxin contamination and improving food security.

## Introduction

Rice, maize, millet, sorghum and wheat are staple cereal crops grown in diverse agroecological zones and consumed by people with varying preferences and socio-economic backgrounds in sub-Saharan Africa [2]. The agricultural sector contributes 20–25% to Côte d'Ivoire’s gross domestic product and employs about 48% of the population [3]. The country’s economy is heavily dependent on the export of cash crops such as cocoa, coffee and cashew nuts, while cereal crops play a crucial role in food security and support the local economy [3]. However, cereal crops are susceptible to post-harvest contamination by a group of filamentous fungi that produce mycotoxins, which pose a threat to human and animal health. For example, *Fusarium* toxins produced by *Fusarium graminearum*, *Fusarium verticillioides* and *Fusarium culmorum* and aflatoxins produced by *Aspergillus flavus* and *Aspergillus parasiticus* are regularly implicated in cereal contamination, both in the field and during storage [4, 5]. In addition to adverse effects on human and animal health [6], mycotoxin contamination of cereal crops results in considerable economic losses and a reduction in the income of farmers and growers [7]. To reduce the burden of post-harvest fungal contamination in modern agriculture, countries with higher socio-economic status rely on the use of synthetic chemicals such as fungicides. However, misuse and overuse of fungicides can be expensive, lead to fungal resistance and cause negative effects on both human and environmental health [8]. Therefore, innovative, eco-friendly and cost-effective solutions are continuously needed to reduce the burden of fungal contamination and to improve the safety of food and agricultural products.

In sub-Saharan Africa, including Côte d’Ivoire, there is long-standing indigenous knowledge of using native plant extracts as an effective solution against plant pests and pathogens [9–14]. Several aromatic plants grown in Côte d’Ivoire have been described to possess medicinal, insecticidal and antimicrobial effects that were used historically by local communities to control post-harvest contamination of food crops [15, 16]. Traditionally, farmers would deploy a crude extract of aromatic plants in granaries during storage to extend the shelf life of cereal crops by preventing fungal contamination and repelling insects, rodents and pests. These plants include *Azadirachta indica* Juss (Neem tree), *Cymbopogon citratus* (DC ex Nees) Stapf. (lemongrass), *Allium sativum* L. (garlic) and *Capsicum frutescens* L. (wild chili pepper) that have been previously reported to have antifungal and antibacterial activities against several human and plant pathogens [17–19]. Additionally, oils extracted from the fresh leaves of these plants possess potent antifungal and antimicrobial activities against *Aspergillus*, *Fusarium* and *Penicillium* spp. [20, 21]. However, essential oil extraction is time-consuming, requires specialized equipment and produces low yields, making the development of oils as fungicides prohibitively expensive for farmers in sub-Saharan countries. Thus, an alternative solution is to explore the ability of an aqueous formulation of native plants to reduce or inhibit fungal contamination of food crops. The use of individual plant extracts offers valuable insights into the specific antifungal mechanism of bioactive metabolites and allows for a more targeted approach against specific micro-organisms. However, mixtures of plant extracts have previously been reported to exhibit combination effects that enhance their bioactive properties [22–25]. Thus, recapitulating traditional knowledge and ancestral practices of using native aromatic plant mixtures for the development of a biofungicide could increase the bioactivity against micro-organisms and the diversity of microbial targets.

BioCC+ is an aqueous extract of plant mixtures of *A. indica* Juss (Neem tree), *C. citratus* (DC ex Nees) Stapf. (lemongrass), *A. sativum* L. (garlic) and *C. frutescens* L. (wild chili pepper) developed from traditional knowledge and ancestral practices of using plants to protect agricultural crops. The constituents of BioCC+ are well-known edible plants in West Africa with very low toxicity ranging between 2,000 mg kg^−1^ body weight for *C. citratus* (lemongrass) and 500 mg kg^−1^ body weight for *A. sativum* (garlic) by oral administration [26, 27]. The objectives of this study were two-fold: first, we employed bioactivity-guided fractionation to compare the efficacy of crude extracts and fractions of BioCC+ against two important cereal pathogens, *F. graminearum* and *F. verticillioides* through *in vitro* antifungal assays. Then, we used untargeted metabolomics and biochemometric approaches to identify bioactive metabolites from the crude extracts and fractions to help explain observed antifungal effects against *Fusarium* spp. Outcomes from this study could provide insights into the antifungal efficacy of BioCC+ and help local researchers and farmers with further research and development of this prototype fungicide for the control of fungal contamination in cereal crops.

## Methods

### Development and preparation of BioCC+

The polyherbal formulation BioCC+ is an aqueous formulation developed by the research team of Dr Elisee Kouassi Kporou (co-first author) at Jean Lorougnon Guede University, Côte d’Ivoire, inspired by Ivorian ancestral practices of using plant mixtures for crop protection. BioCC+ is a mixture of plant material harvested from local medicinal plants: leaves of *A. indica* and *C. citratus*, bulbs of *A. sativum* and fruits of *C. frutescens*. Plant material was macerated together in distilled water for 48 h at room temperature (22–23 °C) with shaking, filtered and oven-dried for 4 days at 40 °C. The dry extract, herein referred to as native crude extract (NCE), was packaged in plastic jars and stored at 4 °C until use.

### *Fusarium* strains used in this study

*F. graminearum* (CB-CS-5) and *F. verticillioides* (61-MS-CS) were provided by Dr Gretchen Kuldau (The Pennsylvania State University) for this study. Fungal spores were obtained by plating *Fusarium* isolates onto sporulation medium, synthetic nutrient-poor agar (1 g l^−1^ KH_2_PO_4_, 1 g l^−1^ KNO_3_, 0.5 g l^−1^ MgSO_4_·7H_2_O, 0.5 g l^−1^ KCl, 0.2 g l^−1^ glucose, 0.2 g l^−1^ sucrose, 20 g l^−1^ agar). Inoculated plates were incubated at 28±2 °C for 7 days. Spores were harvested using 0.05% Tween-20 and stored in 25% glycerol at −80 °C with a concentration of 10^6^ spores ml^−1^. Identity of fungal strains was confirmed by PCR amplification followed by Sanger sequencing of the ITS1/ITS4 region using primers (ITS1F: 5′-TCCGTAGGTGAACCTGCGG-3′ and ITS4: 5′-TCCTCCGCTTATTGATATGC-3′) prior to use [28].

### Antifungal activity assay

Antifungal activity assays were conducted on *F. graminearum* and *F. verticillioides* in 24-well microplates using a range of concentrations for NCE, chemical extracts and fractions (detailed description below) dissolved in DIFCO^™^ Potato Dextrose Agar (PDA) (BD, Franklin Lakes, NJ, USA), to a final volume of 2 ml/well containing fungal growth medium with test compounds. DMSO (2%) (Sigma-Aldrich, St. Louis, MO, USA) was used as a vehicle for chemical extracts and fractions. The range of concentrations tested was guided by antifungal efficacy (percent growth inhibition of test condition compared to control). Therefore, the range for NCE, methanol extract (MeOHe) and H_2_Oe was 3.0 to 25.0 mg ml^−1^, DCMe was between 0.8 mg ml^−1^ and 6.0 mg ml^−1^, and fractions were evaluated between 0.2 mg ml^−1^ and 2.0 mg ml^−1^.

A 1 µl spore suspension containing 10⁵ spores ml^−1^ (equivalent to 100 spores/well) of each *Fusarium* strain was centre-inoculated in each test well and incubated at 30 °C for 3 days. For each test compound and control, four wells were used, and the experiment was repeated twice (two independent biological experiments) for each concentration range (total, *n*=8). Controls for each condition included an uninoculated PDA, PDA with no antifungal treatment inoculated with fungal isolates and PDA+DMSO vehicle inoculated with fungi. Fungal colony diameter was used to measure growth inhibition, and the MIC was defined on day 3 as the lowest concentration that completely inhibits fungal growth within the range of tested concentrations and conditions. Percent growth inhibition was calculated using the following formula: Growth Inhibition (%) = [(Dc - Dt) / Dc] × 100, where: Dc=colony diameter of the control and Dt=colony diameter of the treatment.

### Bioactivity-guided fractionation of BioCC+

The organic solvents dichloromethane (DCM), methanol (MeOH) and all other chemicals used for extraction and fractionation were ACS-grade and purchased from Sigma-Aldrich (St. Louis, MO, USA). NCEs were sequentially extracted with DCM, MeOH and distilled water (dH_2_O) in order of increasing polarity (Fig. S1, available in the online Supplementary Material). Initially, 20 g of NCE was macerated in 250 ml DCM for 48 h while stirring at room temperature (22–23 °C). Then, the macerate was filtered and evaporated to obtain the DCM extract (DCMe). The residue was subsequently extracted using 250 ml MeOH to obtain a MeOH extract (MeOHe). Finally, a water extract (H_2_Oe) was obtained by the addition of 250 ml dH_2_O from the residue of MeOH extraction. This fractionation procedure was performed six independent times, each starting from a defined mass (20 g) of the same crude aqueous BioCC+ extract. Extracts obtained from these six independent fractionation experiments were subsequently dried and pooled by solvent phase before antifungal activity assays and metabolomic analysis. Each extract was tested for antifungal activity.

Based on the observed growth inhibition of DCMe against *Fusarium* strains, DCMe was selected for further separation using silica gel column chromatography using the following mixtures of hexane:ethyl acetate as the mobile phase: 100 : 0 for F1, 75 : 25 for F2, 50 : 50 for F3, 25 : 75 for F4 and 0 : 100 for F5, as well as 100% MeOH for F6 (Table S1). The solvents were removed from each fraction using a rotary evaporator. The residue of each fraction was weighed and stored at 4 °C until use. Of the six fractions, five with the most distinct Thin Layer Chromatography profiles (F2, F3, F4, F5 and F6) were selected for antifungal activity assays against *Fusarium* strains and subsequent analysis by Liquid Chromatography-MS (LC-MS).

### Liquid chromatography mass spectrometry analysis

Native crude extract, DCMe and fractions (F2, F3, F4, F5 and F6) were selected for analysis using a Vanquish UHPLC system coupled to an Exploris 120 mass spectrometer (Thermo Fisher Scientific, Waltham, MA, USA) with a heated electrospray ionization source at the Metabolomics Core Facility at The Pennsylvania State University. Separation was achieved on a Waters Acquity CSH C18 column (2.1×100 mm, 1.7 µm particle size), maintained at 40 °C. The mobile phase consisted of solvent A (LC-MS-grade water with 0.1% formic acid) and solvent B (LC-MS-grade acetonitrile with 0.1% formic acid). The gradient elution programme was as follows: 0–2 min at 3% B, 12 min at 40% B, 15 min at 85% B, 21 min at 100% B and 25–30 min at 3% B. The flow rate was 0.25 ml min^−1^ with a 2 µl injection volume. Data were acquired in both positive and negative ionization modes at a resolution of 120,000 and over a scan range of 80–1000 Da. Ion spray voltages were set at 4,000 V for positive mode and −2,600 V for negative mode. Additional parameters included a sheath gas pressure of 35 Arb, auxiliary gas pressure of 10 Arb, sweep gas pressure of 1 Arb, a vaporizer temperature of 150 °C and an ion transfer tube temperature of 325 °C.

## LC-MS data processing

### Raw data processing

Raw MS files (.raw) were processed using MS-DIAL version 5 for automatic peak detection using parameters for Exploris 120 [29]. Peak detection parameters included a minimum peak height of 10^4^ amplitude, an MS tolerance of 0.005 Da and a retention time range of 0–30 min. Using MS-DIAL, peak spectra were detected, deconvoluted, annotated and aligned. To ensure accurate peak detection, peaks were filtered against blanks, with tentative annotations assigned to all tested features using the MS-DIAL library files under the following criteria: a minimum MS1 spectrum match of 65% and at least 3 MS2 spectra match. The resulting aligned peak table, containing mass to charge (*m/z*) values, MS2 spectra, RT, peak areas and tentative annotation, was exported into an Excel file (.xls) (deposited into ScholarSphere data repository [1]). Metabolites detected in the blank samples were subtracted from the tested samples. The characterization of F1 was not included in antifungal activity assays and biochemometric analysis due to its low extracted mass.

### Chemometric and biochemometric analysis of fractions

To differentiate the fraction with the highest antifungal activity (F4) against all other fractions tested, we combined multivariate and correlation-based statistical analyses to help characterize the underlying chemical differences between the most antifungal fraction from others. Annotated spectral features (unique *m/z*_RT pairs) and corresponding peak areas were used for all analyses and performed in RStudio version 2024.09.0+375 [30]. The identified compounds that resulted from these analyses are here referred to as ‘putative antifungal candidates’ and described as compounds that may contribute to the higher growth inhibition and low MIC of F4 of BioCC+ against *F*. *graminearum* and *F. verticillioides*.

Principal component analysis (PCA) was conducted as an unsupervised approach to compare the metabolomic profiles and visualize natural clustering patterns among fractions F2–F6. Partial least squares discriminant analysis (PLS-DA) was performed to discriminate metabolomic profiles of the fractions. Due to a high variable-to-sample ratio of our dataset, the PLS-DA model and Spearman correlation were used for ranking rather than predictive classification of features. The PLS-DA model ranking was validated by a significant permutation test based on the between- to within-group variance ratio (*P*=0.001, n=999 permutations). To further prioritize compounds that were more abundant in the fraction with the highest growth inhibition, fold-change analysis was performed by comparing the abundance in F4 against the maximum abundance observed across all other fractions.

For biochemometric integration, growth inhibition data were combined with the metabolomic dataset for F2–F6. *F. graminearum* was selected for correlation analysis as it exhibited the highest susceptibility against the tested fractions when compared to *F. verticillioides*. Fraction concentrations were standardized at 0.5 mg ml^−1^ for F2, F3, F5 and F6, while F4 was tested at 0.375 mg ml^−1^, corresponding to the lowest concentration at which complete growth inhibition was observed. This adjustment was made due to a lack of overlapping concentrations with F4. Spearman correlation analysis was then performed to assess relationships between metabolite abundance and percent growth inhibition.

### Ranking of putative antifungal candidates

Putative antifungal compounds were prioritized using a multi-criteria ranking approach applied to individual spectral features. Feature prioritization integrated three complementary criteria. First, features were ranked by PLS-DA using the variable importance in projection (VIP) scores, retaining those with VIP >1.0 and highest abundance in F4. Second, features were filtered based on relative enrichment, requiring at least a twofold higher abundance in F4 compared to other fractions (FC >2). Third, features were assessed for their association with percent growth inhibition using Spearman correlation, retaining those with *rho* >0.8. Features meeting all three criteria were considered high-ranking candidates that could be prioritized for further experimental validation. These putative candidates were additionally compared against published literature for reported antifungal activity, providing guidance for feature prioritization. The ranked feature lists for all analyses in both positive and negative ion modes are provided on ScholarSphere for future follow-up studies [1].

## Results and discussion

### Comparing the antifungal efficacy between NCE, chemical extracts and fractions of BioCC+ against *Fusarium* species

**Fig. 1.**
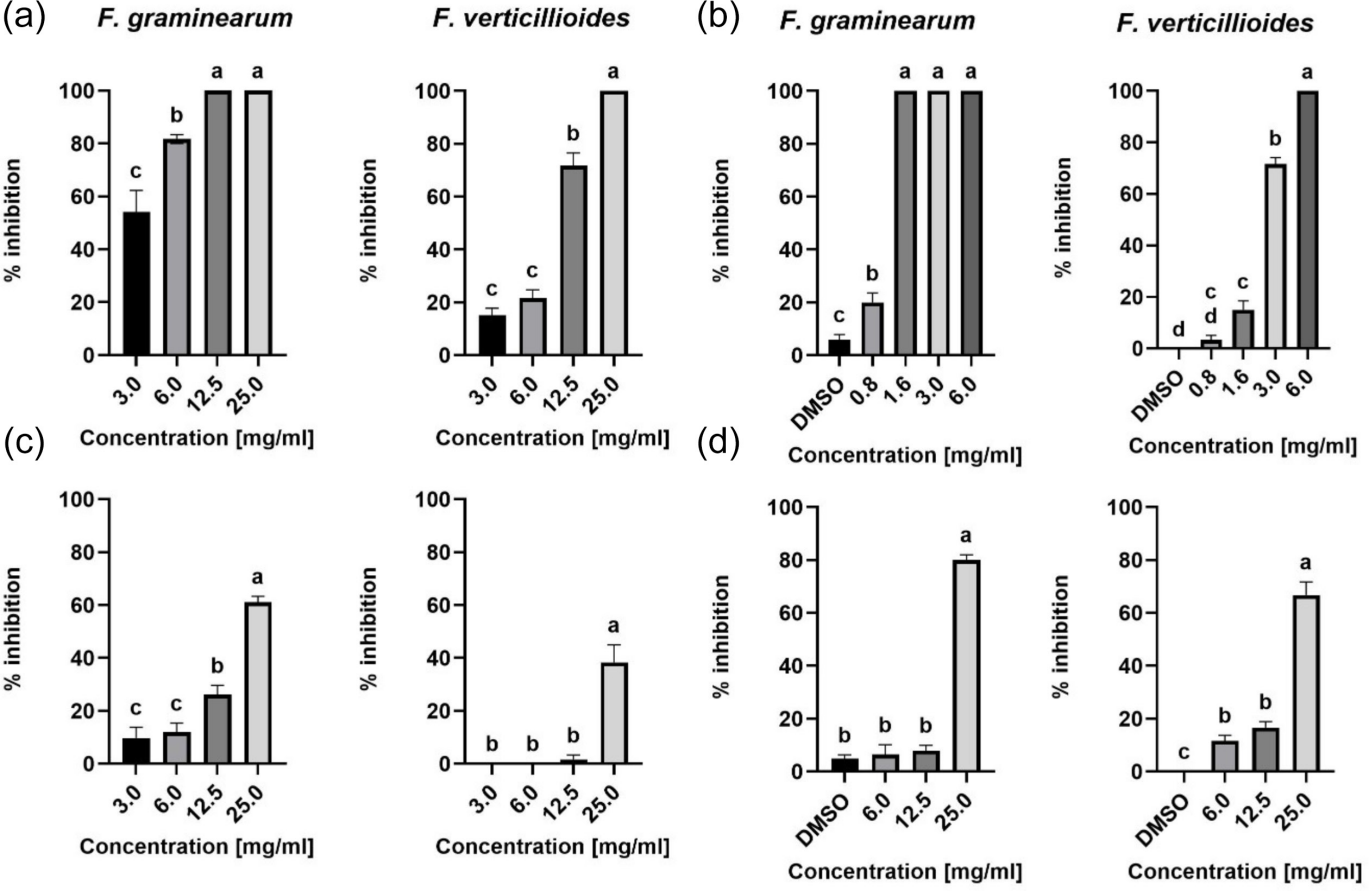
The effect of BioCC+ NCE (**a**), DCMe (**b**), H2Oe (**c**) and MeOHe (**d**) on growth of *F. graminearum* (left column) and *F. verticillioides* (right column). Growth was measured by colony diameter after 72 h and presented as % growth inhibition relative to 0 mg/ml control or DMSO vehicle control (2%) with mean±sem (*n*=8) across two independent experiments. Letters above bar graphs indicate statistical significance using one-way ANOVA analysis followed by Tukey’s multiple comparison test, *P*<0.05.

NCEs of BioCC+ demonstrated dose-dependent antifungal activity against *Fusarium* strains. Complete growth inhibition (100%) was observed at 12.5 mg ml^−1^ with *F. graminearum* and 25.0 mg ml^−1^ with *F. verticillioides*, indicating that *F. graminearum* was more susceptible to BioCC+ NCE compared to *F. verticillioides* (Fig. 1a). Solvent partitions of the NCE were used to isolate bioactive compounds from the aqueous BioCC+ NCE. Antifungal activity assays demonstrate that the DCMe exhibited strong, dose-dependent antifungal activity, achieving 100% growth inhibition of *F. graminearum* at 1.6 mg ml^−1^ and *F. verticillioides* at 6.0 mg ml^−1^ (Fig. 1b). In contrast, neither the H_2_Oe nor MeOHe achieved complete growth inhibition of both *Fusarium* strains within the tested concentration range (Fig. 1c, d).

**Fig. 2.**
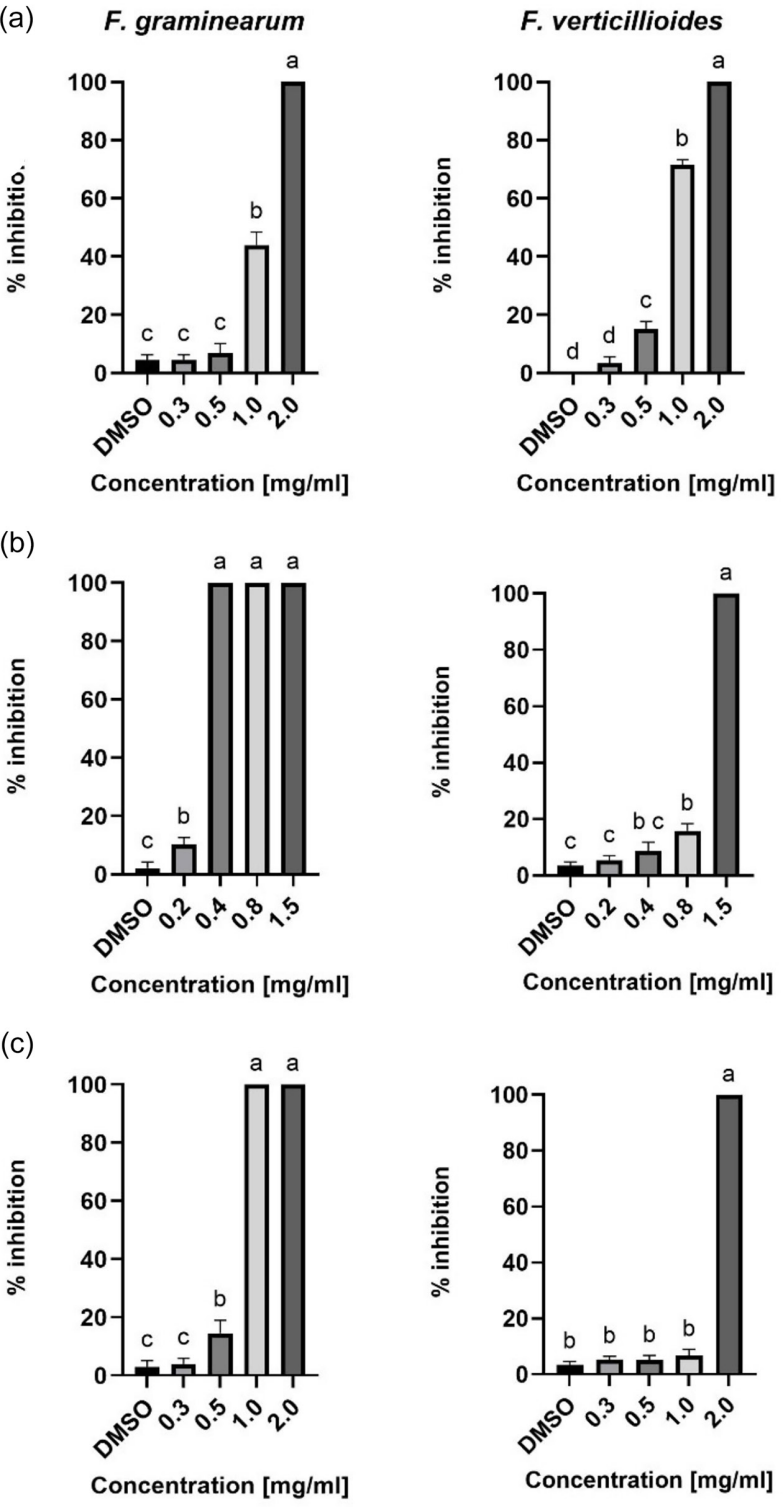
Effect of the most bioactive fractions on growth of *F. graminearum* (left column) and *F. verticillioides* (right column). Growth of *Fusarium* spp. was measured using colony diameter and presented as % growth inhibition relative to the DMSO vehicle control (2%) for F3 (panel a), F4 (panel b) and F6 (panel c). Bar graphs represent mean±sem of % growth inhibition (*n*=8) across two independent experiments. Letters above bar graphs indicate statistical significance using one-way ANOVA analysis followed by Tukey’s multiple comparison test, *P*<0.05.

To investigate the relationship between specific compounds in the DCMe and their antifungal properties, DCMe was further fractionated using silica gel chromatography resulting in six fractions. Among these fractions (F), F3, F4 and F6 exhibited the highest antifungal activity against both *Fusarium* strains (Fig. 2). We observed complete growth inhibition of *F. graminearum* at 2.0 mg ml^−1^, 0.4 mg ml^−1^ and 1.0 mg ml^−1^ for F3, F4 and F6, respectively (Fig. 2). Similar to the antifungal activity of NCE and chemical extracts, *F. verticillioides* was more resistant to the antifungal effects of the tested fractions. Fractions F2 and F5 did not demonstrate significant antifungal activity compared to F3, F4 and F6 (Fig. S2). The MICs for all tested fractions compared to chemical extracts and NCE are summarized in Table S2.

While DCM was selected in the present study for its efficiency in recovering medium- to low-polarity metabolites to help explain which metabolites drive antifungal activity, its volatility and environmental toxicity limit agricultural application. This DCM extract was primarily used for analytical purposes, rather than a formulation intended for field use. From a feasibility standpoint, several strategies can enhance the sustainability and applicability of BioCC+. The crude extract of BioCC+ may be used directly, or the material may be re-extracted or reformulated using alternative solvents such as ethanol, ethyl acetate or ethanol mixtures, which offer a broad polarity range and are approved for agro-industrial applications [31]. Another strategy is to optimize formulation by incorporating the active fractions or purified compounds into biocompatible carriers (e.g. emulsifiable concentrates, microencapsulated or nanoparticle-based systems) that improve stability and delivery for intended use [32]. Collectively, these strategies would enable translation of these putative antifungal compounds from analytical extracts to environmentally sustainable BioCC+-derived biocontrol formulations suitable for agricultural deployment.

Overall, F4 had the highest antifungal efficacy against *F. graminearum* (MIC=0.4 mg ml^−1^) compared to *F. verticillioides* (MIC=1.5 mg ml^−1^). The observed susceptibility of *F. graminearum* compared to *F. verticillioides* could be attributed to physiological differences between the fungal isolates at the tested conditions, though the precise mechanism remains to be investigated. Notably, *F. verticillioides* has been reported to grow more rapidly and produce more toxic secondary metabolites at 30 °C than *F. graminearum* [33]. A relevant future study would be to test the antifungal efficacy of BioCC+ and its chemical extracts and fractions with *Fusarium* isolates grown at various temperatures and on diverse growth media. This could provide insights into mechanistic differences of growth inhibition between *F. graminearum* and *F. verticillioides*.

### Metabolomic profiling of DCM fractions derived from BioCC+

Using MS-DIAL, raw chromatograms from positive and negative modes for all 6 fractions resulted in 22,416 and 11,212 identified features (defined as unique *m/z*_RT pairs), respectively [1]. The MS-DIAL spectral library files were used to tentatively annotate features, where 59% of detected features were annotated in positive mode, while 40% of the detected features were annotated in the negative mode. Here, we focused on annotated features for downstream metabolomic characterization and identification of putative antifungal compounds driving observed growth inhibition of the most bioactive fractions.

**Fig. 3.**
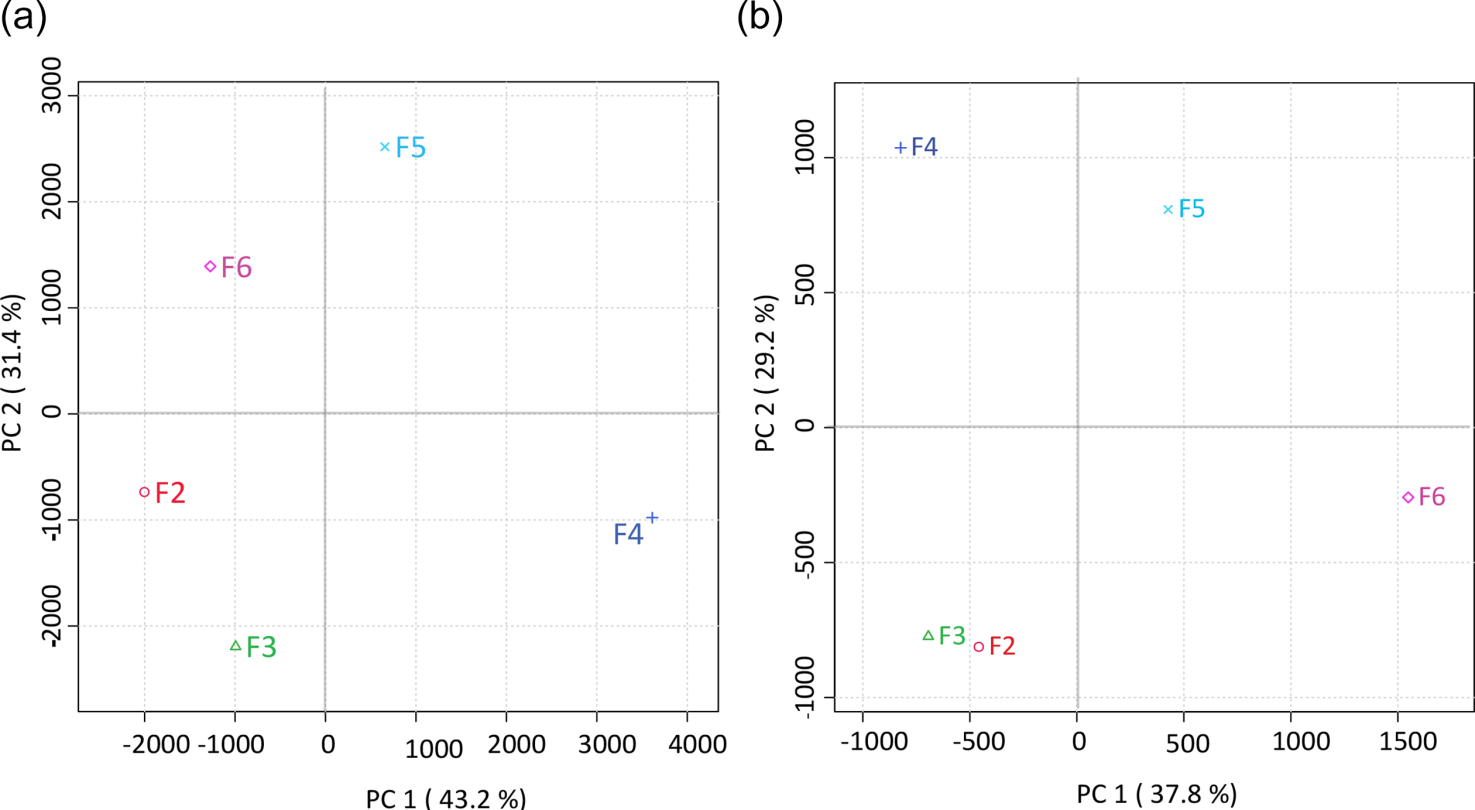
PCA scores plot of the metabolomic profiles from fractions (F2, F3, F4, F5 and F6) in positive (**a**) and negative (**b**) mode using MetaboAnalyst 6.0.

To assess similarities and differences in chemical profiles (chemometrics) of bioactive fractions, PCA was conducted using the peak areas of the annotated features. The top two components of the PCA accounted for 74.6 and 67% of the total variance in positive and negative modes. For both modes, F2 and F3 appear to cluster together along PC1, suggesting similarities in their respective chemical profiles. All other fractions were distinctively different from each other, with F4 having the highest distinction in positive ion mode, while F6 had the highest distinction along PC1 (Fig. 3). The clear metabolomic separation of F4 and F6 from other fractions aligns with their observed high percent growth inhibitions, suggesting that variation in their chemical composition could help explain observed antifungal properties.

### Characterization of putative antifungal candidates from DCM fractions of BioCC+

**Fig. 4.**
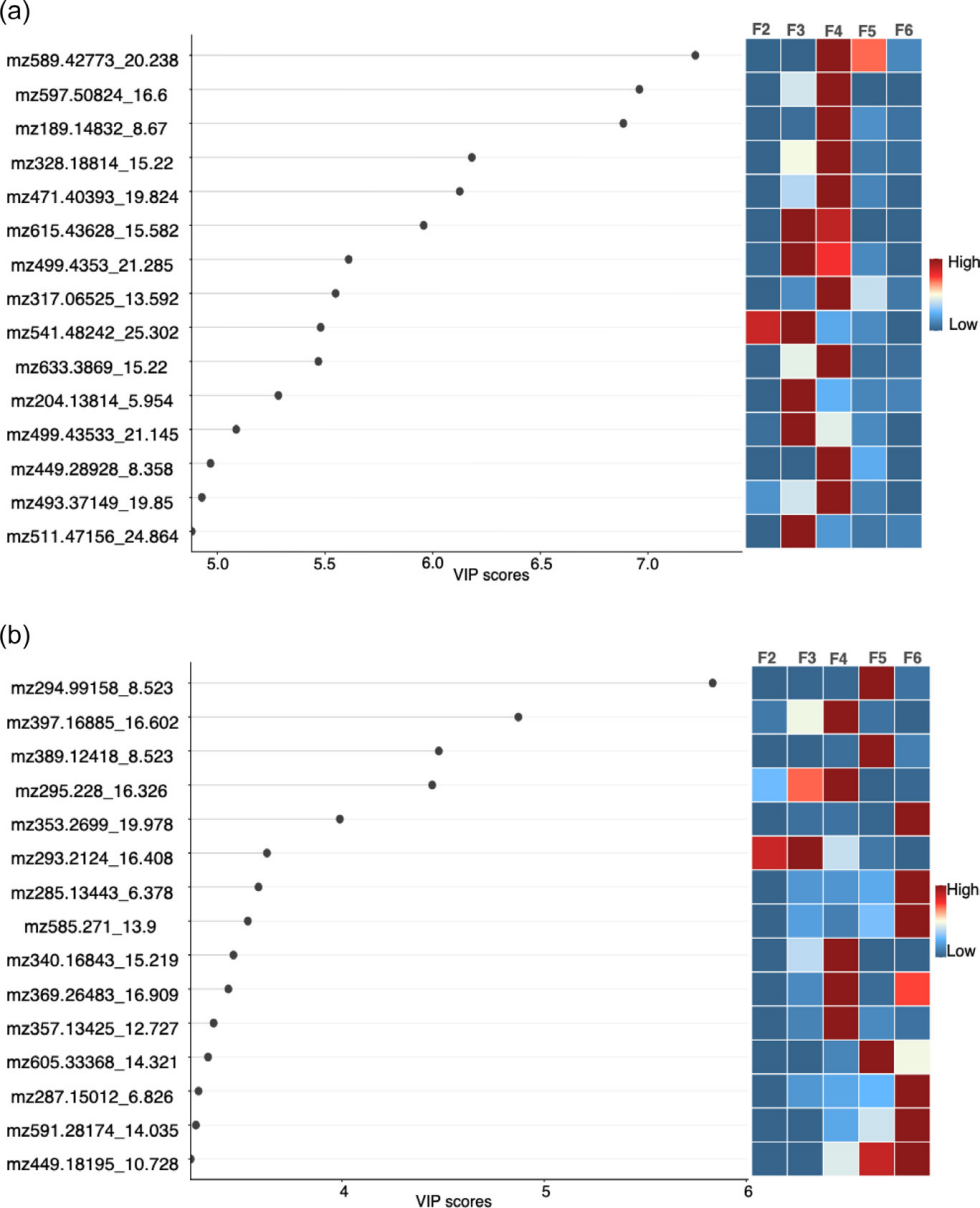
The VIP score plots from PLS-DA with the top 15 highest VIP features identified in the bioactive fractions F2 through F6, both in the (**a**) positive and (**b**) negative mode. The y-axis represents annotated features, and the x-axis shows VIP scores. Feature peak areas were log-transformed and mean-centred. Dark blue to dark red boxes represent metabolite peak areas ranging from lowest to highest, respectively.

PLS-DA was used to identify metabolite features associated with chemical differences among fractions. Model robustness was supported by permutation testing (*P*=0.001, *n*=999), reducing the likelihood of overfitting. Features with VIP scores (VIP >1.0) were considered priority. Among these, those with higher abundance in F4, the fraction with the greatest antifungal activity, were prioritized as putative contributors (Fig. 4). In total, 1,675 discriminatory features were identified in positive mode and 279 in negative mode. Several high-ranking features corresponded to compounds previously reported to exhibit antifungal activity against *Fusarium* spp. [27, 34–36]. An example is capsaicin (mz304.19202_15.224) and chrysoeriol (mz301.07056_13.303), supporting the biological relevance of the model. Complementary analyses were used to further refine candidate features. Fold-change analysis identified 4,171 and 1,043 features total with 673 and 293 features exclusively in F4 in positive and negative ion modes, respectively. Finally, Spearman correlation linked metabolite abundance with percent growth inhibition across fractions. This correlation-based analysis identified 114 and 18 features with high correlation to growth inhibition in positive and negative mode, respectively. Complete feature tables can be found in ScholarSphere [1].

To prioritize putative antifungal candidates from BioCC+, we integrated results from the three statistical analysis approaches (PLS-DA, fold-change and Spearman correlation) and selected features consistently identified across all methods. This consensus strategy resulted in 17 high-confidence candidate features (15 in positive mode, 2 in negative mode [1]). To further increase confidence in compound annotation, these candidate features were cross-referenced with GNPS annotations. This resulted in a refined list of six putatively annotated metabolites (Tables 1 and 2). Synthetic compounds and primary metabolites were excluded from further consideration. Of these six candidates, two have been previously reported to exhibit antifungal activity and were categorized as flavonoids and alkaloids (Tables 1 and 2). These compound classes are broadly associated with antioxidant, anticancer, antibacterial and antifungal properties, supporting the hypothesis that the identified metabolites may contribute to the antifungal effects observed in BioCC+ fractions [37, 38].

**Table 1.** Putative antifungal candidates based on chemometrics and biochemometrics profiling of fractions in positive ion mode

Feature (m/z_RT)	Adduct type	Putative ID	MS/MS spectrum	Class	Reported antifungal activity*	Ref.
mz313.06052_5.746	[M+Na]+	(+)Catechin	61.03168 : 4022 159.98157 : 7383 170.03851 : 5226 179.98730 : 3772 182.99785 : 10883 185.55949 : 3735 198.03351 : 28625 208.01814 : 4422 215.97224 : 5870 216.04486 : 4765 226.02942 : 13389 244.03847 : 9071 254.02426 : 10667 272.03476 : 9816 290.04437 : 17801 313.06042 : 12153	Flavonoid	Yes But could also possess some phytotoxicity to some plant roots.	[39, 44]
mz160.03276_7.809	[M+Na]+	Trigonelline†	55.93444 : 11138 116.97195 : 21347	Alkaloid	Yes	[41, 42]
mz393.22714_9.306	[M+H]+	(E)-3-(acetyloxymethyl)-5-(2-formyl-4-hydroxy-5,5,8a-trimethyl-1,4,4a,6,7,8-hexahydronaphthalen-1-yl)pent-2-enoic acid	107.61997 : 3506 116.17297 : 3630 135.11728 : 13669 170.62646 : 3913 179.10710 : 19519 197.11728 : 115925 284.27014 : 4090 320.64502 : 4030 393.22760 : 7306	Diterpenoid	Unknown	
mz292.99805_10.416	[M+H]+	1,2-Didehydrotanshinone IIA	null	Diterpenoid	Yes to similar structured compounds	[45]

*Column ‘Antifungal’ refers to annotated antifungal activity based on published literature: ‘Yes’ denotes published literature with demonstrated antifungal activity of the tentatively identified compound through* in vitro* or *in vivo *studies; ‘Unknown’ denotes that no studies were found that specifically tested the compound for its antifungal properties.

†Refers to compounds whose names, m/z and RT, have been putatively identified using GNPS library in addition to MS-DIAL.

‘null’ refers to compounds with no found fragment ions of the parent compound using MS-DIAL.

m/z_RT refers to mass to charge_retention time of the compounds.

MS/MS spectrum refers to fragment ions of the tentatively identified compounds.

**Table 2.** Putative antifungal candidates based on chemometrics and biochemometrics profiling of fractions in negative ion mode

Feature (m/z_RT)	Adduct type	Putative ID	MS/MS spectrum	Class	Reported antifungal activity*
mz327.25415_19.306	[M-H]-	2,4-Dihydroxyheptadec-16-enyl acetate	89.02545 : 4076 193.49608 : 4437 206.62772 : 4068 215.65144 : 4152 242.98553 : 24353 255.23306 : 438856 262.99387 : 4615 286.99622 : 4384 326.99454 : 16492 327.18158 : 4486 327.25497 : 9773	Lipid/fatty acyl	Unknown
mz811.46448_20	[M+HCOO]-	Cauloside C	121.06588 : 10723 143.47859 : 4616 152.37431 : 4121 188.93207 : 4099 193.08763 : 6124 257.28659 : 4597 281.24908 : 77431 284.40451 : 4719 362.14395 : 4483 483.20160 : 6746 542.84064 : 4469 542.97668 : 5207 725.87927 : 5934 765.47131 : 5675 811.03723 : 12666 811.46045 : 25946	Triterpenoid saponin	Unknown

m/z_RT refers to mass to charge_retention time of the compounds.

MS/MS spectrum refers to fragment ions of the tentatively identified compounds.

* Column ‘Antifungal’ refers to annotated antifungal activity based on published literature. ‘Unknown’ denotes that no studies were found that specifically tested the compound for its antifungal properties.

Some putatively identified metabolites have prior literature support for antifungal activity, supporting the biological relevance of the chemometric prioritization. For example, (+) catechin is a flavan-3-ol polyphenol with demonstrated antifungal activity against clinically relevant *Candida* strains through intracellular reactive oxygen species accumulation and plasma membrane disruption [39]. Catechin and its epimer epicatechin were the major constituents of the n-butanol fraction of the *Pinus wallichiana* leaf extracts which completely inhibited the growth of *Fusarium oxysporum* at 40 mg ml^−1^ *in vitro* [40]. Another compound is trigonelline, an alkaloid that has been shown to inhibit *Candida albicans* at an MIC of 4 µg ml^−1^ [41]. Trigonelline is also a major constituent of fenugreek seed ethanolic extracts, which controlled *F. graminearum* at an MIC of 500 mg ml^−1^ *in vitro* [42].

In addition, some candidates share structural similarity with bioactive compound classes such as terpenoids. Of note, cauloside C shares structural similarities with cauloside A, which has been reported to reduce fungal pathogen diseases, such as rice blast, tomato leaf blight and tomato grey mould up to 97% at 500 µg ml^−1^ [43]. Taken together, the presence of multiple compounds from known bioactive classes suggests that the antifungal activity observed in F4 is likely driven by the combined or synergistic effects of multiple metabolites rather than a single compound. However, targeted isolation and validation will be required to confirm their individual and combined roles. Another limitation of this study is the lack of true biological replicates (independently grown or harvested plant material or multiple batches of BioCC+), limiting the statistical and interpretative power of our metabolomics data. The decision to rely on technical replication and fraction-based comparisons rather than independent biological replicates was driven by (i) biomass and resource constraints associated with producing sufficient quantities of BioCC+ extract for fractionation, antifungal screening and metabolomic profiling, and (ii) precedent in published metabolomics studies that successfully integrate fractionation-based designs with multivariate analyses to link chemical features to biological activity. Future studies should incorporate fully independent biological replicates to strengthen the robustness of metabolite–antifungal activity associations.

Using biochemometrics that combines antifungal activity and metabolomic profiling, our study demonstrates that plant mixtures of BioCC+, a formulation traditionally developed based on ancestral practices in Côte d'Ivoire, is effective in inhibiting the growth of two major fungal pathogens, *F. graminearum* and *F. verticillioides*. Integration of bioactivity assays with chemometric data using multiple statistical analysis models helps explain the observed efficacy of BioCC+ components and facilitates the ranking and discovery of novel compounds for potential development of new biofungicides. Outcomes from this study could provide insights into sourcing more local plant extracts to further improve the strength and specificity of the prototype biofungicide by integrating antifungal efficacy with metabolite fingerprinting.

## Supplementary material

10.1099/acmi.0.001173.v3 Supplementary Material 1.

## Abbreviations

DCM: dichloromethane
DCMe: dichloromethane extract
FC: fold change
H2Oe: water extracts
LC-MS: Liquid Chromatography-MS
MeOH: methanol
MeOH: methanol
MeOHe: methanol extract
MeOHe: methanolic extract
NCE: native crude extract
PCA: principal component analysis
PDA: potato dextrose agar
PLS-DA: partial least squares discriminant analysis
VIP: variable importance in projection
