# Supplementary material for: Evaluating the antifungal efficacy and bioactive metabolites of a traditional polyherbal aqueous formulation BioCC+ against Fusarium graminearum and Fusarium verticillioides
Source: Access Microbiol. 2026 Aug 11;8(8):001173.v3. doi: 10.1099/acmi.0.001173.v3 (PMC13463721; doi:10.1099/acmi.0.001173.v3)
Supplement: Supplementary file 1 [file acmi-8-01173-s001.pdf]

Supplementary Material for Review

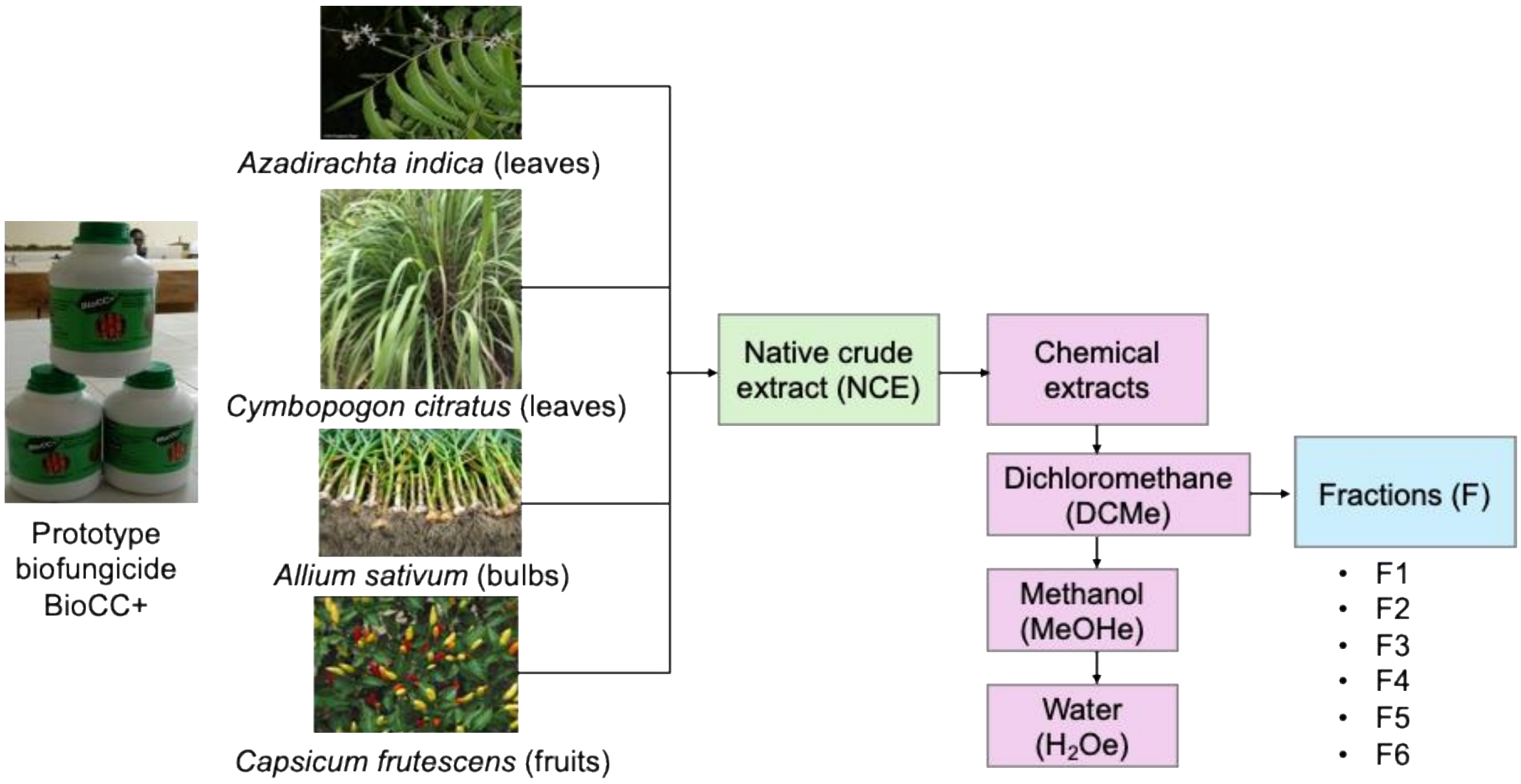

Figure S1 Schematic diagram of extraction and bioactivity-guided fractionation to evaluate the antifungal properties of the polyherbal formulation, BioCC+ against *F. graminearum* and *F. verticillioides*. The green box denotes NCE, three chemical extracts (DCMe, MeOHe, H_2Oe) are indicated in pink boxes, and six fractions (F) are indicated in blue.

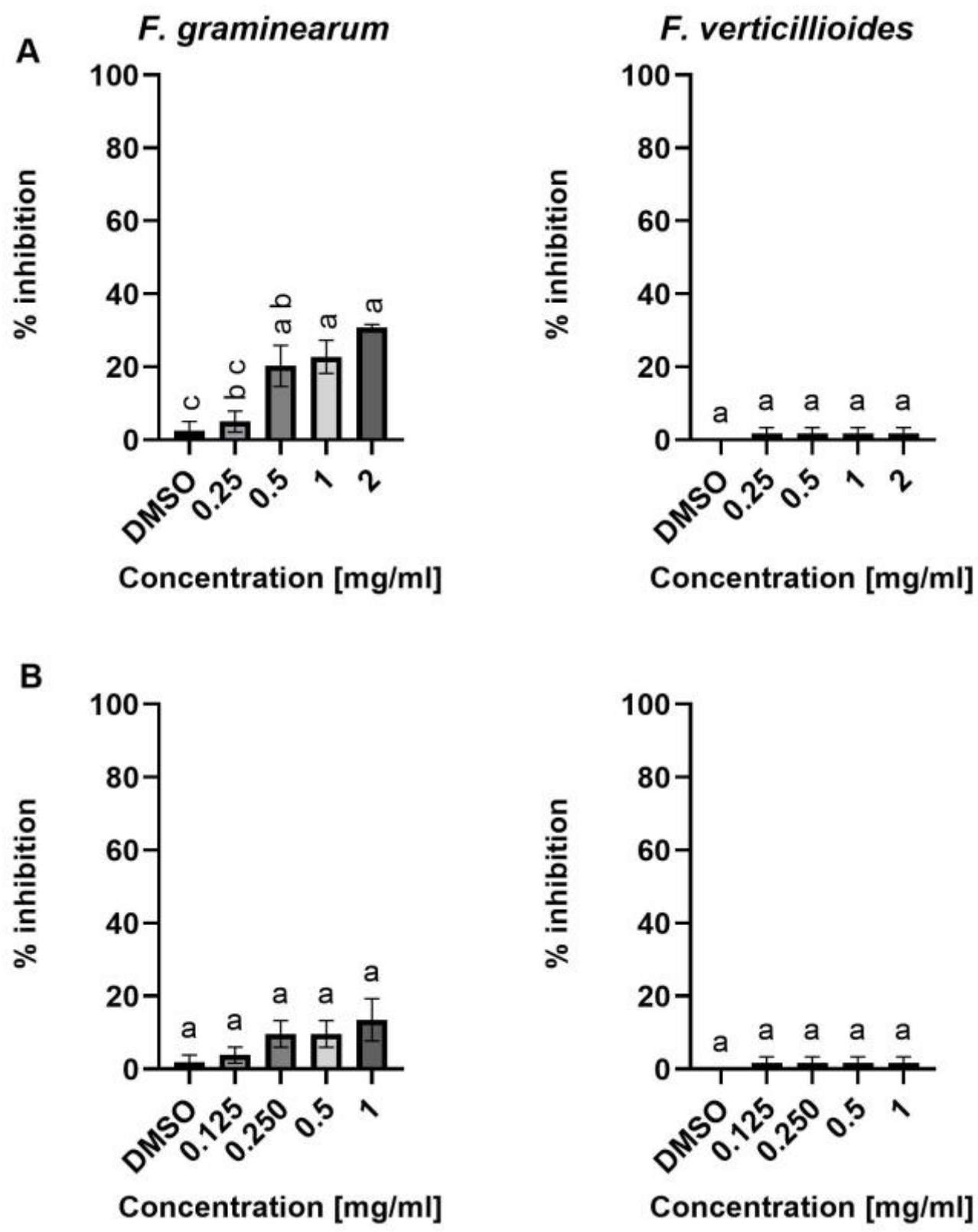

Figure S2 The effect of fractions F2 and F5 on growth of *F. graminearum* (left column) and *F. verticillioides* (right column). Growth of *Fusarium* spp. was measured using colony diameter and presented as % growth inhibition relative to the DMSO vehicle control (2%) for F2 (panel A) and F5 (panel B). The bar graphs represent mean ± SEM of % growth inhibition (n=4). Letters above bar graphs indicate statistical significance using one-way ANOVA analysis followed by Tukey's multiple comparison test, P<0.05.

Table S1 Mobile phase composition used for fractionation of DCM extracts using column chromatography

Mobile phase	Proportions V/V	Fraction	Mass (mg)	Yield (%)
Hexane-Ethyl acetate	100/0	F1	10	1.42
Hexane-Ethyl acetate	75/25	F2	280	40
Hexane-Ethyl acetate	50/50	F3	100	14.2
Hexane-Ethyl acetate	25/75	F4	60	8.57
Hexane-Ethyl acetate	0/100	F5	40	5.72
Methanol	100	F6	210	30

V/V : volume to volume ratio

Table S2 Summary of MIC values for BioCC+, chemical extracts, and fractions against *Fusarium* spp. grown on PDA at 30°C for 72 h

Species tested	MIC (mg/mL)									
	BioCC+	DCM	MeOH	H2O	F1	F2	F3	F4	F5	F6
F. graminearum	12.5	1.6	>25.0	> 25.0	NT	>2.0	2.0	0.4	>1.0	1.0
F. verticillioides	25.0	6.3	> 25.0	> 25.0	NT	NT	2.0	1.5	NT	2.0

NT : Not tested
